# The emerging role of ISWI chromatin remodeling complexes in cancer

**DOI:** 10.1186/s13046-021-02151-x

**Published:** 2021-11-04

**Authors:** Yanan Li, Han Gong, Pan Wang, Yu Zhu, Hongling Peng, Yajuan Cui, Heng Li, Jing Liu, Zi Wang

**Affiliations:** 1 Department of Hematology, Institute of Molecular Hematology, The Second Xiangya Hospital, Central South University, Changsha, 410011 Hunan China; 2 Molecular Biology Research Center and Hunan Province Key Laboratory of Basic and Applied Hematology, School of Life Sciences, Central South University, Changsha, 410078 Hunan China

**Keywords:** ISWI family, Cofactors, Transcription complexes, Tumor immunology, Inhibitors

## Abstract

Disordered chromatin remodeling regulation has emerged as an essential driving factor for cancers. Imitation switch (ISWI) family are evolutionarily conserved ATP-dependent chromatin remodeling complexes, which are essential for cellular survival and function through multiple genetic and epigenetic mechanisms. Omics sequencing and a growing number of basic and clinical studies found that ISWI family members displayed widespread gene expression and genetic status abnormalities in human cancer. Their aberrant expression is closely linked to patient outcome and drug response. Functional or componential alteration in ISWI-containing complexes is critical for tumor initiation and development. Furthermore, ISWI-non-coding RNA regulatory networks and some non-coding RNAs derived from exons of ISWI member genes play important roles in tumor progression. Therefore, unveiling the transcriptional regulation mechanism underlying ISWI family sparked a booming interest in finding ISWI-based therapies in cancer. This review aims at describing the current state-of-the-art in the role of ISWI subunits and complexes in tumorigenesis, tumor progression, immunity and drug response, and presenting deep insight into the physiological and pathological implications of the ISWI transcription machinery in cancers.

## Background

Normal gene transcription is fundamental for cell physiology. The gene transcription program is executed by transcription complexes (TCs). Chromatin remodeling complexes (CRCs) are multisubunit TCs containing a series of ATP-dependent remodeling enzymes, which act as ‘molecular motors’ that couple ATP hydrolysis to the perturbation of histone-DNA contacts with respect to individual nucleosome core particles [1]. Based on the sequence homology of the catalytic ATPase and the accessory subunits, CRCs are divided into four main subfamilies: switch/sucrose nonfermentable (SWI/SNF), chromodomain-helicase DNA-binding protein (CHD), inositol-requiring mutant 80 (INO80) and imitation switch (ISWI) [1–3]. Generally, ISWI complexes help the initial histone–DNA complexes (pre-nucleosomes) to mature into canonical octameric nucleosomes and spacing of nucleosomes at relatively fixed distances [4, 5], and are involved in multiple aspects of cell physiology, such as transcriptional regulation [6–9], DNA damage response, repair and recombination [10–13].

To date, a growing number of preclinical and clinical studies have highlighted that ISWI complexes play critical pathological roles in tumorigenesis, tumor development, tumor immunity and drug response. ISWI subunits display multiple functions in affecting tumor cell phenotypes via regulation of oncogenic gene transcription. Somatic mutations, copy number changes or translocations have been identified that may produce gain/loss-of-function properties of ISWI subunits. Deregulation of ISWI complexes by abnormal expression or activity disrupts the normal interplay between ISWI subunits and TFs or facilitates the activity of oncogenic ISWI-containing TCs, which is expected to upset gene regulatory networks. Here, we provide unique insight into the implications of ISWI complexes and subunits in cancer.

### The ISWI complex: types and composition

**Fig. 1:**
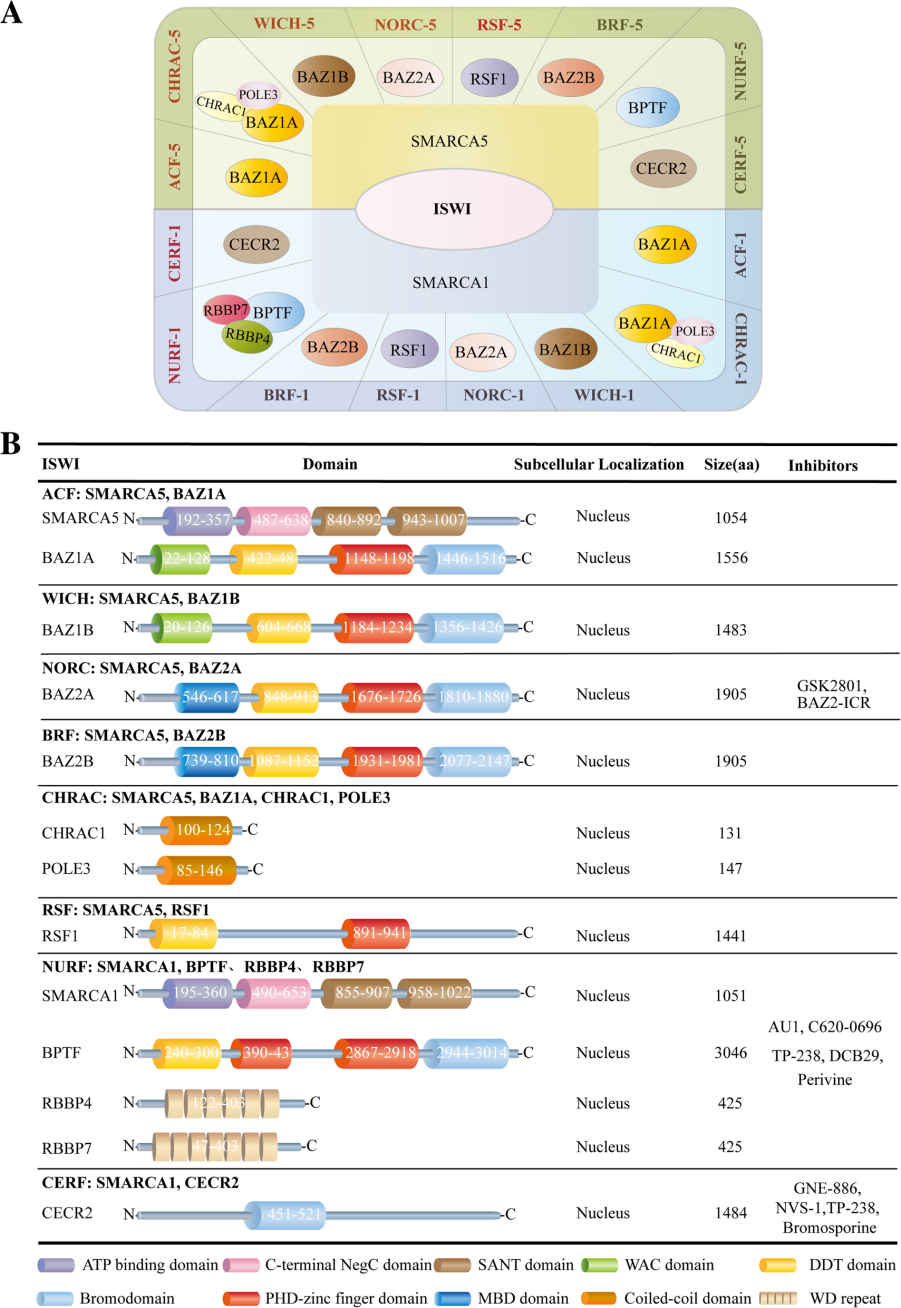
Classification of ISWI family. Molecular components, functional domains, subcellular localization and targeting inhibitors for the ISWI family. **A** Sixteen different types of ISWI complexes are shown [14, 22, 48]. They harbor either SMARCA1 or SMARCA5 as ATPase subunits and 1–3 noncatalytic subunits. **B** Schematic representation of functional domains, protein subcellular localization, number of nucleotides and targeting inhibitors for each ISWI protein member

ISWI family is one of the best conserved ATPase families. It possesses highly conserved SWI2/SNF2 family ATPase domain, belonging to the superfamily of DEAD/H-helicases, that provides the motor for chromatin remodeling and a characteristic HAND-SANT-SLIDE domains with DNA binding activity [4]. Chromatin remodeling complexes containing the ISWI ATPase, including NURF, CHRAC and ACF, were, originally identified in Drosophila homologs, and later shown to be highly conserved in many other organisms, such as yeast and mammals [14]. In general, the CHRAC and ACF complexes seem to function in assisting nucleosome sliding [15]. NURF acts particularly in the epigenetic regulation, such as the regulation of higher-order chromatin structure [4]. The ISWI complexes display various variants over different species. For example, there are two ISWI catalytic subunit variants (Isw1 and Isw2) in *Saccharomyces cerevisiae*, forming 4 different complexes via association with different subunits [16]. Isw1 forms an Isw1a complex with Ioc3, which prevents the binding of basal Pol II transcription machinery to the promoter and inhibits transcription initiation [16]. In addition, Isw1 forms an Isw1b complex together with Ioc2 and Ioc4 subunits, which play a regulatory role in Pol II transcription elongation and termination [4]. Isw2 forms a complex with Itcl, Dpb4 and Dls1, which regulates the spacing of nucleosome series and play a remodeling function [4]. In *Drosophila*, it contains only one ISWI ATPase, which is a constituent of three complexes: dNURF, dACF and dCHRAC [17]. dNURF promotes H1 loading onto chromosomes in vivo and directly facilitated some genes-mediated transcription from chromatin templates, such as GAL4 [4, 18]. dNURF can be recruited by the transcriptional repressor dKen to repress STAT responsive-genes, blocking activation until signal thresholds are reached [19]. dACF is able to assist the assembly of chromatin with regular nucleosome spacing and is capable of catalyzing considerable ACF-dependent motility of entire chromatosomes within fully loaded arrays [20]. In mammals, a specific ISWI complex is composed of one ATPase subunit (SMARCA5 or SMARCA1) and one to three noncatalytic subunits [21]. Specially, both human ATPases, SMARCA1 and SMARCA5, form stable complexes with all regulatory subunits, which expands the ISWI complex members up to 16, including RSF-1/RSF-5 (SMARCA1/5 and RSF1), ACF-1/ACF-5 (SMARCA1/5 and BAZ1A), CHRAC-1/CHRAC-5 (SMARCA5/1, BAZ1A, CHRAC1 and POLE3), WICH-1/WICH-5 (SMARCA1/5 and BAZ1B), NoRC-1/NoRC-5 (SMARCA1/5 and BAZ2A), NuRF-1/NuRF-5 (SMARCA1/5, BPTF, RBBP7 and RBBP4), CERF-1/CERF-5 (SMARCA1/5 and CECR2) and BRF-1/BRF-5 (SMARCA1/5, BAZ2B) [14, 22] (Fig. 1A). Different subunits possess different functional domains and play distinct roles in complexes, which is summarized in Fig. 1B and Table 1.

**Table 1:** Functional domains of ISWI proteins

Domain	Functions
ATP binding domain	ATP binding domain, an autonomous nucleosome remodeling machine, interacts with the super helical location 2 (SHL2) of the nucleosomal DNA, with the N-terminal tail of H4 and with the α1 helix of H3
C-terminal NegC domain	The C-terminal NegC domain is involved in binding to the core2 domain and functions as an allosteric element for ISWI to respond to the extranucleosomal DNA length
SANT domain	SANT domain has a central role in chromatin remodeling by functioning as a unique histone-interaction module that couples histone binding to enzyme catalysis, and it is important for nucleosome sliding activity, such as the regulation of nucleosome spacing
WAC domain	WAC domain is involved in the interaction of ACF with chromatin and the binding of other ACF-related factors to DNA
DDT domain	DDT domain associates with the histone modifications H3K4me3 and H4K16ac and facilitates DNA binding
Bromodomain	Bromodomain is a conserved motif, which recognizes acetylated lysine residues of histones or interacting proteins
MBD domain	The MBD (methyl-CpG-binding) domain specifically recognizes and binds to methylated CpGs. This binding allows it to trigger methylation of H3K9 and results in transcriptional repression
PHD-type zinc finger domain	PHD-type zinc finger domain binds specific epigenetic marks on histone tails to recruit transcription factors and nucleosome-associated complexes to chromatin. For example, it resides in the BPTF subunit of NURF, interacts directly with H3K4me3, stabilizes association of BPTF/NURF with chromatin

### Genetic alterations in ISWI members are common in cancer and correlated with prognosis

**Fig. 2:**
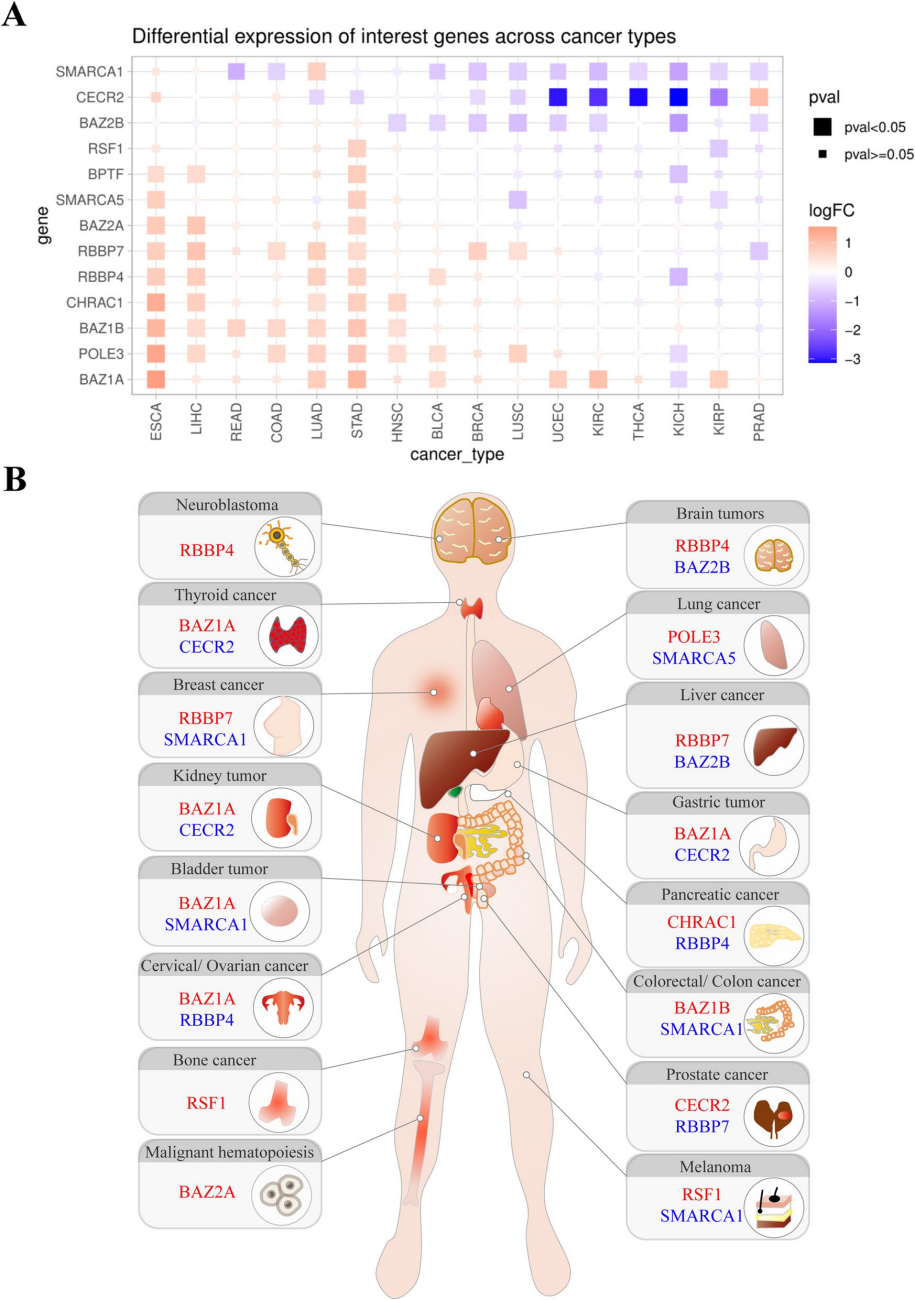
Gene expression analysis of ISWI family members in human cancers. **A** Fold changes in gene expression of ISWI members across a variety of tumor types compared to the normal control, based on the TCGA dataset (only tumor types with more than 10 normal samples and tumor samples were selected). FPKM data were used as the expression profile, and the R language limma package was used to conduct variance analysis. Red indicates upregulation, and blue indicates downregulation (*P* < 0.05 after correction) in tumors. LogFC = log2 (average (tumor)/average (normal)). Full names for cancer abbreviations are shown in the abbreviation table. **B** A schematic diagram shows the most significant change in the mRNA expression levels of ISWI member genes in different types of cancer based on TCGA dataset analysis or literature summaries. The most significantly upregulated or downregulated genes are marked in red and in blue, respectively

The pan-cancer analysis of TCGA dataset and a growing number of studies revealed that ISWI subunits are abnormally expressed in human cancers (Fig. 2A and B). Whole genome, exome and transcriptome sequencing identified aberrant genetic states for ISWI subunits, including somatic mutations, abnormal copy numbers and gene fusions in various tumor types (Tables 2, 3 and 4). The genetic abnormality is a main factor determining the levels of some ISWI subunits in a particular type of cancer, and contributing to tumor phenotypes. For example, *BPTF* gene copy number is frequently amplified in human tumors, particularly in melanoma [23], neuroblastomas and lung cancers [24]. Specifically, in 67% of the BPTF-positive lung tumors cases, gain of the 17q24.3 locus was associated with poor prognosis (grade III). Knock-down of excessive BPTF negates the pre-malignant phenotype of highly proliferating lung embryonic fibroblasts cells [24].

**Table 2:** ISWI subunits with high frequency mutations in malignancies

Tumor	Gene	Mutation number	Case number with mutation	Percentage (total number)
Acinar Cell Carcinoma of the Pancreas	RBBP4	2	2	8.6%(23)
	BPTF	4	3	13%(23)
Acral Melanoma	RSF1	1	1	10%(10)
Adenoid Cystic Carcinoma	BAZ2B	2	2	5.3%(38)
Angiosarcoma	BPTF	3	3	3.6%(83)
Basal Cell Carcinoma	BAZ2B	15	15	5.1%(293)
	BAZ2A	18	14	4.8%(293)
	BAZ1A	14	13	4.4%(293)
	BAZ1B	13	13	4.4%(293)
	RSF1	9	9	3.1%(293)
Bladder Cancer	BAZ2B	29	25	6.1%(412)
	BPTF	26	24	5.1%(474)
	RSF1	18	17	4.1%(412)
	BAZ2A	17	16	3.9%(412)
Cervical Squamous Cell Carcinoma	BPTF	13	12	4.1%(291)
Cholangiocarcinoma	SMARCA1	1	1	12.5%(8)
Colon Cancer	BPTF	9	9	8.5%(106)
	SMARCA1	30	26	4.9%(534)
Colorectal Adenocarcinoma	SMARCA1	30	26	4.9%(534)
	BPTF	26	26	4.2%(619)
	BAZ2B	26	20	3.7%(534)
	RSF1	29	19	3.6%(534)
	BAZ1A	25	19	3.6%(534)
	BAZ1B	22	19	3.6%(534)
	BAZ2A	21	19	3.6%(534)
	RBBP7	18	16	3.0%(534)
Cutaneous Squamous Cell Carcinoma	BAZ2B	14	13	33.3%(39)
	BAZ2A	8	8	20.5%(39)
	BAZ1A	8	5	12.8%(39)
	SMARCA1	4	3	7.7%(39)
	SMARCA5	3	3	7.7%(39)
	RSF1	4	3	7.7%(39)
	BAZ1B	3	3	7.7%(39)
Desmoplastic Melanoma	BPTF	3	3	15%(20)
Esophageal Carcinoma	BPTF	36	35	6.8%(518)
	BAZ2B	25	22	4.2%(518)
	BAZ2A	20	18	3.5%(518)
	SMARCA1	103	56	10.8%(517)
Gallbladder Carcinoma	BAZ2B	2	2	6.3%(32)
	BPTF	2	2	6.3%(32)
Gastric Cancer	CECR2	3	3	3.8%(78)
	BAZ2B	5	5	3.4%(147)
Head and Neck Squamous Cell Carcinoma	BAZ2B	18	17	3.3%(515)
Intrahepatic Cholangiocarcinoma	BPTF	7	7	6.8%(103)
Liver Hepatocellular Carcinoma	BAZ2B	3	3	6.5%(46)
	BPTF	12	12	3.2%(373)
Lung Adenocarcinoma	BAZ2B	13	12	6.7%(179)
	BAZ1A	11	9	5.0%(179)
	BAZ2B	11	11	4.8%(230)
	BPTF	9	8	4.4%(183)
	SMARCA1	7	7	3.9%(179)
	SMARCA1	21	20	3.5%(566)
	BAZ1B	7	6	3.4%(179)
Lung Squamous Cell Carcinoma	BAZ2B	28	27	5.6%(484)
	BPTF	21	19	3.9%(484)
	BAZ1B	16	15	3.1%(484)
Lung Squamous Cell Carcinoma	SMARCA1	9	9	3.9%(179)
Mantle Cell Lymphoma	BPTF	1	1	3.4%(29)
Metastatic Melanoma	BPTF	22	17	11.8%(144)
	BAZ2A	14	14	9.7%(144)
	BAZ1A	8	8	5.6%(144)
Non-Hodgkin Lymphoma	RBBP4	1	1	7.1%(14)
	BPTF	18	18	3.6%(500)
Pleural Mesothelioma	BPTF	1	1	4.5%(22)
Primary Central Nervous System Lymphoma	SMARCA5	1	1	10%(10)
Prostate Cancer	CHRAC1	1	1	3.3%(30)
Skin Cutaneous Melanoma	BPTF	52	48	10.9%(440)
	BAZ2A	47	40	9.1%(440)
	BAZ1A	36	26	5.9%(440)
	RSF1	26	25	5.7%(440)
	SMARCA1	23	23	5.2%(440)
	BAZ1B	20	20	4.5%(440)
	RBBP4	15	13	3%(440)
Small-Cell Lung Cancer	BPTF	9	9	7.5%(120)
	RSF1	7	7	5.8%(120)
Stomach Adenocarcinoma	BPTF	34	30	7.6%(395)
	BAZ1A	16	16	4.1%(395)
	RSF1	16	15	3.8(395)
	BAZ2A	14	14	3.5%(395)
Urothelial Carcinoma	BPTF	5	5	6.9%(72)
	RSF1	1	1	6.3%(16)
Uterine Clear Cell Carcinoma	BAZ1B	1	1	6.3%(16)
	CECR2	3	3	4.2%(72)
Uterine Corpus Endometrial Carcinoma	SMARCA1	103	56	10.8%(517)
	BPTF	93	52	10.1%(517)
	BAZ1A	70	46	8.9%(517)
	SMARCA5	64	42	8.1%(517)
	BAZ2A	62	40	7.7%(517)
	RSF1	56	38	7.4%(517)
	BAZ1B	68	38	7.4%(517)
	RBBP7	30	23	4.4%(517)
	RBBP4	18	16	3.1%(517)

Data come from the TCGA database

**Table 3:** ISWI subunits with high frequence of abnormal copy numbers in malignancies

Tumor	Gene	Cytoband	Type of CNA	Case number with CNA	Percentage (total number)
Acral Melanoma	RSF1	11q14.1	AMP	6	15.8%(38)
	BAZ2A	12q13.3	AMP	2	5.3%(38)
Adenoid Cystic Carcinoma	BAZ2A	12q13.3	HOMDEL	6	10%(60)
	BAZ1A	14q13.1-q13	HOMDEL	4	6.7%(60)
Adrenocortical Carcinoma	BAZ2A	12q13.3	AMP	3	3.3%(90)
	POLE3	9q32	AMP	3	3.3%(90)
Adult Soft Tissue Sarcomas	CHRAC1	8q24.3	AMP	9	4.4%(206)
Angiosarcoma	RBBP4	1p35.1	AMP	3	3.6%(83)
	BPTF	17q24.2	AMP	3	3.6%(83)
	CHRAC1	8q24.3	AMP	3	3.6%(83)
Bladder Cancer	CHRAC1	8q24.3	AMP	21	5.1%(408)
	RSF1	11q14.1	AMP	16	3.9%(408)
	BAZ2B	2q24.2	AMP	5	1.1%(442)
Brain Lower Grade Glioma	CHRAC1	8q24.3	AMP	18	3.5%(511)
Breast Cancer	CHRAC1	8q24.3	AMP	449	20.7%(2173)
	RSF1	11q14.1	AMP	204	9.4%(2173)
	BPTF	17q24.2	AMP	167	7.7%(2173)
	BAZ1A	14q13.1-q13.2	AMP	10	4.2%(237)
	CECR2	22q11.1-q11.21	HOMDEL	10	4.2%(237)
	RBBP4	1p35.1	AMP	7	3%(237)
Colon cancer	BPTF	17q24.2	HOMDEL	8	7.6%(105)
	BAZ2B	2q24.2	AMP	2	1.9%(105)
		2q24.2	HOMDEL	1	1.0%(105)
Colorectal Adenocarcinoma	CHRAC1	8q24.3	AMP	21	3.5%(592)
Esophageal Carcinoma	CHRAC1	8q24.3	AMP	21	11.5%(182)
	RSF1	11q14.1	AMP	18	4.8%(378)
	SMARCA5	4q31.21	AMP	6	3.3%(182)
	BPTF	17q24.2	AMP	12	3.2%(378)
	BAZ2B	2q24.2	AMP	2	1.1%(184)
Gastric Cancer	SMARCA1	Xq25-q26.1	HOMDEL	106	98.1%(108)
	RBBP7	Xp22.2	HOMDEL	105	97.2%(108)
	CHRAC1	8q24.3	AMP	22	20.4%(108)
	BAZ1B	7q11.23	AMP	7	6.5%(108)
	BPTF	17q24.2	AMP	5	4.6%(108)
	RSF1	11q14.1	AMP	5	4.6%(108)
	POLE3	9q32	AMP	4	3.7%(108)
Head and Neck Squamous Cell Carcinoma	CHRAC1	8q24.3	AMP	41	7.9%(517)
	RSF1	11q14.1	AMP	18	3.5%(517)
Liver Hepatocellular Carcinoma	CHRAC1	8q24.3	AMP	60	16.2%(370)
	BPTF	17q24.2	AMP	16	4.3%(370)
	BAZ2B	2q24.2	AMP	4	1.1%(370)
Lung Adenocarcinoma	BAZ1A	14q13.1-q13	AMP	60	11.6%(516)
	CHRAC1	8q24.3	AMP	15	5.0%(302)
	RSF1	11q14.1	AMP	20	3.9%(516)
	BPTF	17q24.2	AMP	19	3.7%(516)
Lung Squamous Cell Carcinoma	CHRAC1	8q24.3	AMP	36	7.2%(501)
	BPTF	17q24.2	AMP	18	3.6%(501)
	BAZ2B	2q24.2	AMP	4	1.1%(370)
		2q24.2	HOMDEL	8	1.6%(501)
Melanoma	CHRAC1	8q24.3	AMP	17	26.6%(64)
	BAZ1B	7q11.23	AMP	11	17.2%(64)
	BPTF	17q24.2	AMP	5	7.8%(64)
	RSF1	11q14.1	AMP	4	6.3%(64)
	SMARCA5	4q31.21	AMP	2	3.1%(64)
	BAZ2A	12q13.3	AMP	2	3.1%(64)
		12q13.3	HOMDEL	2	3.1%(64)
	POLE3	9q32	AMP	2	3.1%(64)
		9q32	HOMDEL	3	4.7%(64)
	BAZ2B	2q24.2	AMP	3	4.7%(64)
		2q24.2	HOMDEL	1	1.6%(64)
Mesothelioma	BPTF	17q24.2	AMP	5	5.7%(87)
Ovarian Serous Cystadenocarcinoma	CHRAC1	8q24.3	AMP	156	27.3%(572)
	RSF1	11q14.1	AMP	57	10%(572)
	RBBP4	1p35.1	AMP	27	4.7%(572)
Pancreatic Cancer	CHRAC1	8q24.3	AMP	14	12.8%(109)
	BAZ1B	7q11.23	AMP	7	6.4%(109)
	RBBP4	1p35.1	HOMDEL	6	5.5%(109)
	SMARCA5	4q31.21	HOMDEL	6	5.5%(109)
	BPTF	17q24.2	HOMDEL	4	3.7%(109)
	BAZ2B	2q24.2	AMP	2	1.8%(109)
Pediatric Neuroblastoma	BPTF	17q24.2	AMP	2	3.4%(59)
Prostate Cancer	CHRAC1	8q24.3	AMP	87	19.6%(444)
	RBBP7	Xp22.2	AMP	47	10.6%(444)
	BAZ1B	7q11.23	AMP	25	5.6%(444)
	BPTF	17q24.2	AMP	22	5%(444)
	POLE3	9q32	AMP	21	4.7%(444)
	BAZ1A	14q13.1-q13.2	AMP	18	4.1%(444)
	SMARCA1	Xq25-q26.1	AMP	16	3.6%(444)
	BAZ1B	7q11.23	AMP	5	3.3%(150)
	RBBP4	1p35.1	AMP	1	3.3%(30)
Stomach Adenocarcinoma	CHRAC1	8q24.3	AMP	32	7.3%(441)
	RSF1	11q14.1	AMP	16	3.6%(441)
Urothelial Carcinoma	BPTF	17q24.2	AMP	13	24.5%(53)
	CHRAC1	8q24.3	AMP	8	15.1%(53)
	CECR2	22q11.1-q11.21	AMP	3	5.7%(53)
	RSF1	11q14.1	AMP	3	5.7%(53)
	BAZ1A	14q13.1-q13.2	AMP	3	5.7%(53)
	BAZ1B	7q11.23	AMP	2	3.8%(53)
	BAZ2B	2q24.2	AMP	3	5.7%(53)
Uterine Carcinosarcoma	CHRAC1	8q24.3	AMP	5	8.9%(56)
	BPTF	17q24.2	AMP	3	5.4%(56)
	SMARCA1	Xq25-q26.1	HOMDEL	2	3.6%(56)
	BAZ2B	2q24.2	AMP	1	1.8%(56)
Uterine Corpus Endometrial Carcinoma	CHRAC1	8q24.3	AMP	24	4.5%(539)
	BAZ2B	2q24.2	AMP	6	1.1%(539)
Uveal melanoma	CHRAC1	8q24.3	AMP	14	17.5%(80)
Invasive ductal cancer	BAZ1A	14q12-q13	AMP	7	5.74%(122)

All data come from the TCGA database

*HOMDEL* homozygouse deletion, *AMP* amplification

**Table 4:** ISWI subunits with abnormal gene fusions in malignancies

Tumors	Gene	Fusion number	Case number with fusion	Percentage (total number)
Adrenocortical Carcinoma	BAZ1A	1	1	1.1%(90)
Bladder Urothelial Carcinoma	SMARCA5	1	1	0.2%(408)
Brain Lower Grade Glioma	RSF1	1	1	0.2%(511)
Breast Invasive Carcinoma	RSF1	8	8	0.8%(1070)
	RBBP7	2	2	0.2%(1070)
	BAZ2B	1	1	< 0.1%(1070)
	RBBP4	1	1	< 0.1%(1070)
	CHRAC1	1	1	< 0.1%(1070)
Prostate	SMARCA1	2	2	0.2%(494)
Lung Adenocarcinoma	BPTF	1	1	0.2%(511)
Metastatic Solid Cancers	SMARCA1	2	2	0.4%(500)
Ovarian Serous Cystadenocarcinoma	RBBP4	2	2	0.4%(523)
	RSF1	1	1	0.2%(523)
Pancreatic Adenocarcinoma	BPTF	1	1	0.6%(183)
	CHRAC1	1	1	0.6%(179)
Prostate	SMARCA1	2	2	0.2%(494)
Sarcoma	RSF1	2	2	0.8%(253)
	RBBP7	2	2	0.8%(253)
Skin Cutaneous Melanoma	RSF1	4	4	0.9%(440)
	RBBP7	1	1	0.2%(500)
Small-Cell Lung Cancer	RSF1	1	1	0.8%(125)
Uterine Corpus Endometrial Carcinoma	BAZ1B	2	2	0.4%(500)

All data come from the TCGA database

Some ISWI subunits have been found to be closely related to patient prognosis. For example, serum RSF1 DNA levels are obviously upregulated in lung cancer patients and closely associated with TNM stage and lymph node metastasis [25]. In HER^2+^ breast tumors, high levels of BAZ1A are associated with detrimental relapse-free survival (RFS) and extremely poor overall survival (OS) [26]. The *BAZ1A* gene within the 14q12-q13 amplicon is frequently amplified in esophageal squamous cell carcinoma (ESCC), while this region harbors some oncogenic genes whose amplification leads to the development and progression of various types of tumors, such as small cell lung cancer (SCLC) and non-small cell lung cancer (NSCLC) [27]. The levels of BAZ2A are upregulated and associated with poor prognosis and recurrence in various tumors, such as prostate cancer [28]. Interestingly, high levels of BAZ2A correlate with the deletion of the *PTEN* gene in primary prostate cancer, which can be an indicator of poor prognosis [29]. BPTF is suggested to be a pan prognostic marker in various cancers. BPTF, which is highly expressed in NSCLC tumor tissues, is positively associated with advanced clinical stage, more lymph nodes and distant metastasis [30, 31]. In particular, it is strongly relevant to the risk for lung adenocarcinoma (LUAD) with EGFR mutations [32]. Moreover, BPTF, amplified in human breast tumors, is linked to shorter metastasis-free survival (MFS) [33, 34].

### Roles of ISWI subunits and ISWI-containing transcription complexes in cancer development

The ATPase subunit of the major mammalian ISWI complex interacts with different proteins, forming multiprotein complexes that in some cases have many subunits. ISWI ATPases also interact with a variety of DNA-binding factors and cofactors, which are involved in malignant transformation and tumor progression. In contrast, noncatalytic subunits are critical for the functional diversity of ISWI complexes and perform specialized functions in cancers.

### SMARCA1

SMARCA1 is broadly expressed in primary human tissues. However, multiple types of genetic abnormalities, including mutation, amplification and deletion lead to changes in SMARCA1 expression in tumors (Tables 2, 3 and 4). SMARCA1 plays an oncogenic or a tumor suppressor role, depending on tumor type. In breast, lung and cervical cancer, the oncogenic effects of SMARCA1 involve cell survival and cell cycle progression [35]. Inhibition of SMARCA1 increases the activity of caspase 9 by upregulating the expression of Apaf-1, which subsequently activates the caspase cascade [35]. In contrast, as a tumor suppressor, SMARCA1 is frequently silenced in gastric cancer due to aberrant DNA methylation. SMARCA1 knockdown promotes the growth of gastric tumor cells, accompanied by downregulation of genes related to cellular homeostasis [36]. Similarly, SMARCA1 is strongly expressed in normal melanocytes but is widely absent in malignant melanoma. Forced expression of SMARCA1 in melanoma cells inhibits cell proliferation and metastasis by attenuating Wnt/β-catenin signaling [37].

**Fig. 3:**
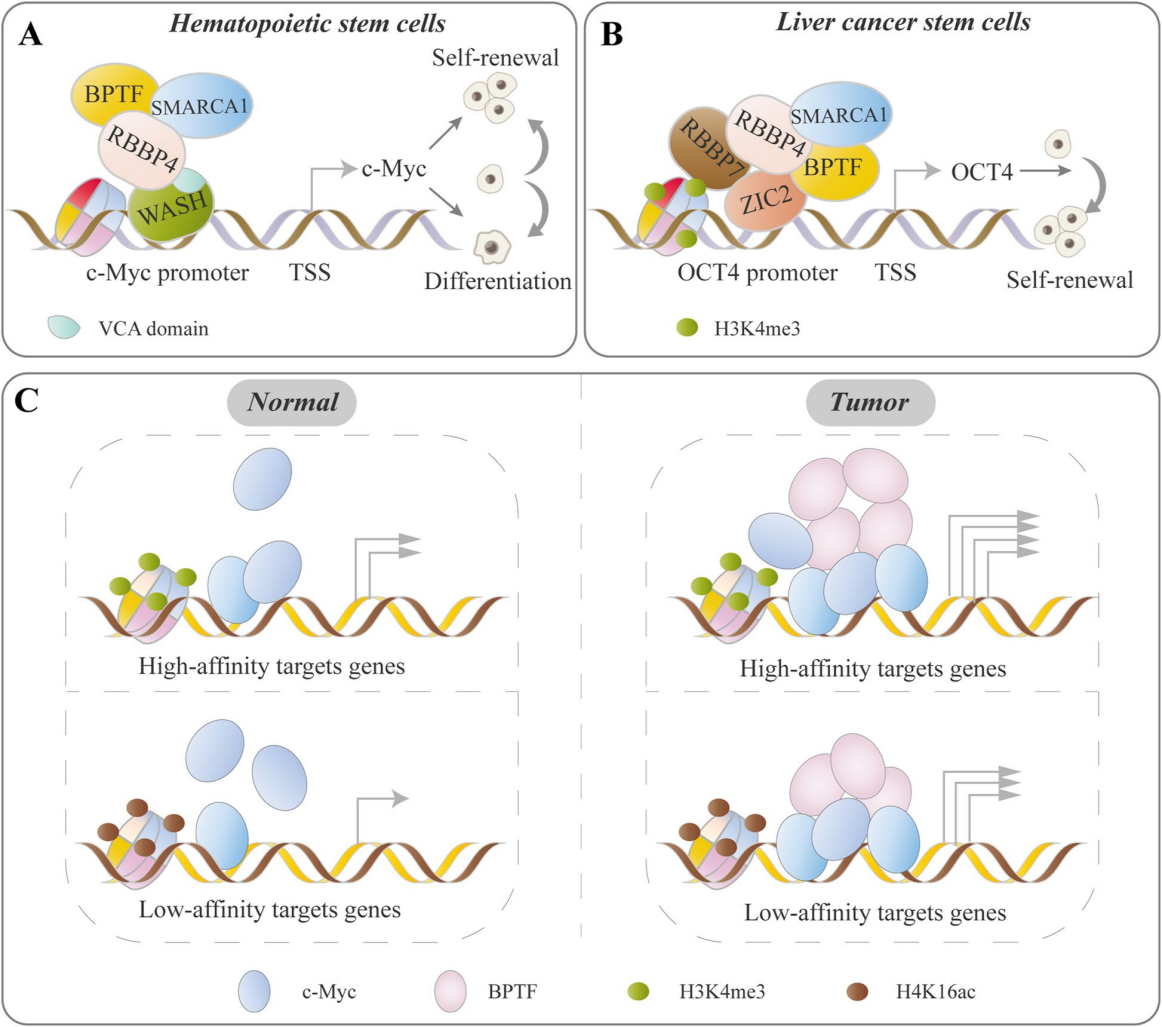
Representative models of ISWI-IF/cofactor interplay. ISWI family proteins regulate cell phenotypes via association with TF/cofactor, in which the ISWI–TF/cofactor interplay is critical for optimal TF activity. **A** WASH recruits the NURF complex to the c*-Myc* promoter through VCA domain-dependent nuclear actin nucleation, which initiates *c-Myc* expression and maintains the self-renewal and differentiation potential of LT-HSCs [38]. **B** In liver CSCs. ZIC2 recruits the NURF complex to the *OCT4* promoter, accompanied by enrichment of H3K4me3 and increased chromatin accessibility of *OCT4*, which activates *OCT4* transcription and drives the self-renewal of liver CSCs [39]. **C** BPTF functions as a crucial cofactor of c-MYC required for tumorigenesis. The BPTF requirement for target recognition by c-MYC depends on the epigenetic context: it is dispensable for c-MYC to bind with H3K4me3-rich ‘high-affinity’ promoters and is also necessary for c-MYC to bind with ‘low-affinity’ sequences. BPTF leads to increased c-MYC recruitment to target DNA and regulates chromatin accessibility at c-MYC target promoters, thus increasing c-MYC target gene transcription and driving tumorigenesis [99–101]

It has been found that SMARCA1 regulates normal stem cell or cancer stem cell (CSC) characteristics by interplaying with other types of cofactors. For example, WASH, an actin nucleating factor of the WASP family, assists the recruitment of SMARCA1-NURF complex to the *c-Myc* promoter and enhances gene transcription through VCA domain-dependent nuclear actin nucleation, which maintains the differentiation potential of long-term hematopoietic stem cells (LT-HSCs) to mature blood lineages [38] (Fig. 3A). Additionally, the SMARCA1-NURF complex was recruited to the *OCT4* promoter by TF ZIC2 and initiated *OCT4* transcription via elevated chromatin accessibility and H3K4 me3 levels of the *OCT4* locus, which maintained the self-renewal of liver CSCs [39]. Correspondingly, high expression of the SMARCA1-NURF complex, ZIC2 and OCT4 are positively correlated with the clinicopathological stages of hepatocellular carcinoma (HCC) [39] (Fig. 3B).

### SMARCA5

SMARCA5 and SMARCA1 share a high degree of amino acid sequence homology but appear to have different functions, as judged, for example, by their expression profiles and involvement in structurally and functionally different remodeling complexes [40]. SMARCA5 is frequently overexpressed in breast cancer [41], ovarian cancer [42], HCC [43] and acute myeloid leukemia (AML) [44, 45]. In breast cancer, the SMARCA5 expression level is positively correlated with tumor size, TNM stage and poor overall survival. Knockdown of SMARCA5 inhibits cell proliferation by arresting the G1 to S phase transition and suppresses MMP2-mediated invasion [41]. In ovarian cancer, the interplay between Rsf-1 and SMARCA5 contributes to tumor cell survival and growth. As a partnership of SMARCA5, induction of Rsf-1 expression not only facilitates translocation of SMARCA5 into nuclei where it colocalizes with Rsf-1 but also enhances SMARCA5 protein expression [42]. In HCC, SMARCA5 is able to promote cell survival and proliferation by increasing the protein level of β-catenin and enhancing its nuclear accumulation [43]. SMARCA5-containing fusion proteins caused by chromosomal translocations also contribute to carcinogenesis. For example, Sumegiet et al. identified that a chromosomal translocation t(4;22)(q31;q12) causes an in-frame fusion of the first 7 exons of EWSR1 to the last 19 exons of *SMARCA5* in extraskeletal Ewing sarcoma/primitive neuroectodermal tumors. NIH3T3 cells expressing the EWSR1–SMARCA5 fusion exhibited anchorage-independent growth and formed colonies in soft agar, indicating this chimeric protein has tumorigenic potential [46].

**Fig. 4:**
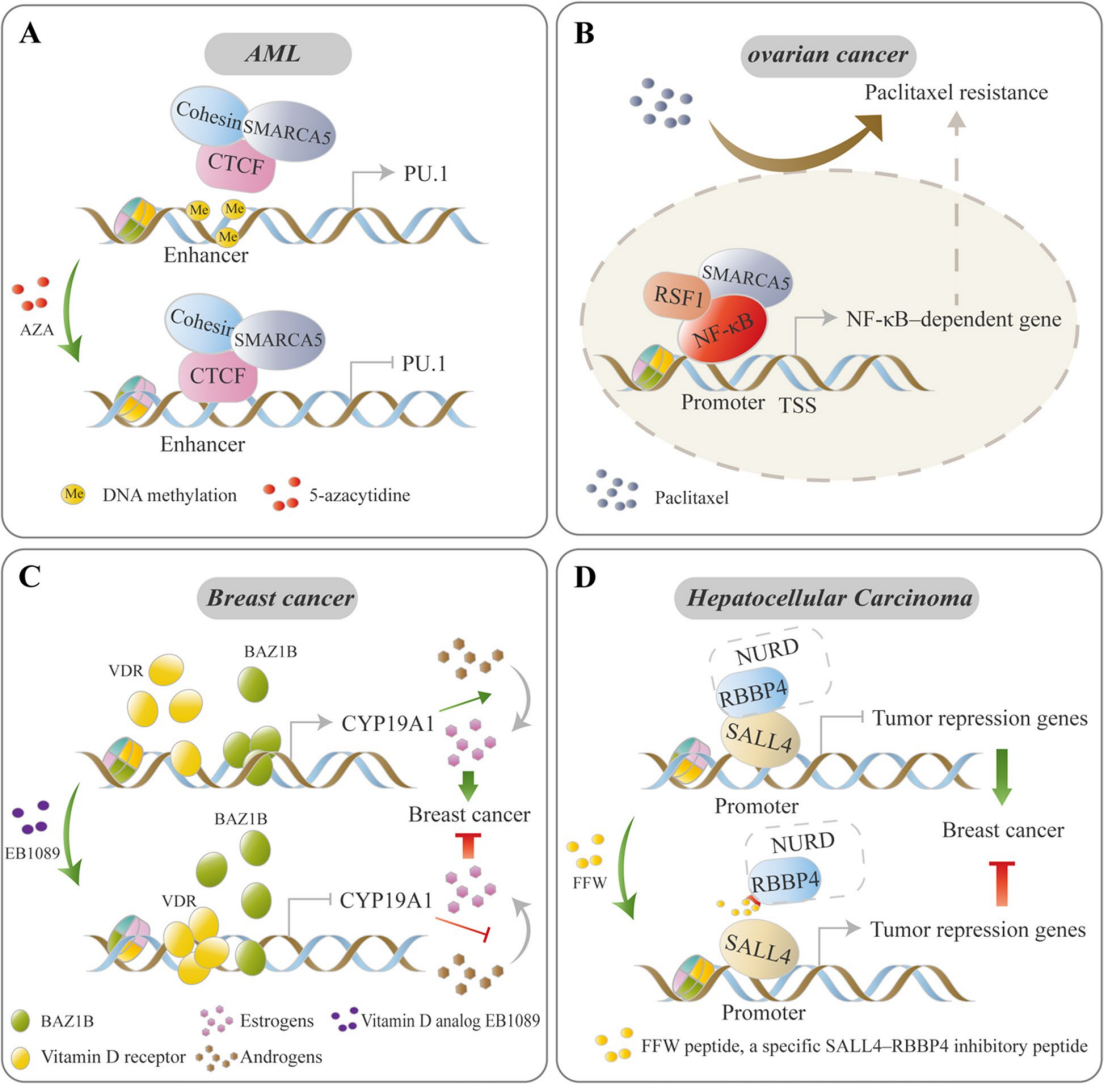
ISWI proteins mediate the chemotherapy response and chemical resistance. Some chemotherapy response- and chemoresistance-related genes are regulated by ISWI complexes. The transition of ISWI occupancy by changing DNA methylation levels or targeting ISWI protein/TF interactions may be two treatment strategies for pharmacological intervention. **A** In AML, the binding of the SMARCA5-CTCF complex at the PU.1 gene is blocked due to DNA methylation. Upon treatment by AZA-mediated DNA demethylation, the SMARCA5-CTCF complex is recruited to the enhancer of the *PU.1* gene and inhibits its expression [45]. **B** The RSF1-SMARCA5 complex functions as a coactivator for NF-κB, consequently augmenting the expression of NF-κB-dependent chemoresistance-related genes, further resulting in paclitaxel resistance in ovarian cancer cells [56]. **C** Breast cancer cells with estrogen receptor positivity. After EB1089 (vitamin D analog) treatment, the occupancy of vitamin D receptor (VDR) and BAZ1B on CYP19A1 (encoding the enzyme aromatase, which can catalyze the conversion of androgens to estrogens) promoter are altered with the recruitment of VDR and dissociation of BAZ1B, which results in the inhibition of CYP19A1 transcription and contributes to the EB1089 treatment response by arresting the transformation of androgens to estrogens [70, 71]. **D** In HCC, the recruitment of the NuRD complex by SALL4 to the promoter of some tumor suppressors via an interaction of SALL4 with RBBP4. FFW (a highly potent SALL4-RBBp4 antagonist peptide) treatment abolishes the binding of SALL4 to RBBP4, which leads to the reactivation of tumor suppressors [111]

SMARCA5 plays an important role in hematological malignancies. SMARCA5 was upregulated in CD34+ AML progenitors, and loss of SMARCA5 inhibited AML cell proliferation [44, 45]. Moreover, the binding site of SMARCA5-CTCF complex at the key hematopoietic regulator *PU.1* was methylated in AML, which blocked the complex binding. Upon demethylation by 5-azacitidine (AZA) treatment, the occupancy of SMARCA5-CTCF complex was partly restored, which led to the downregulation of *PU.1* transcription and induced myeloid differentiation, indicating that the occupancy of the SMARCA5-CTCF complex at the specific hematopoietic gene is critical for normal hematopoiesis [45] (Fig. 4A).

It should be noted that although *SMARCA5* and *SMARCA1* have overlapping expression patterns in some cases, the activities of SMARCA1- and SMARCA5-containing complexes likely are not fully redundant. For instance, the spatial and temporal expression patterns of SMARCA1 and SMARCA5 are distinct throughout murine development and in the adult animals, indicating that they may have non-overlapping functions [40]. Through analysis of TCGA database, the *SMARCA5* and *SMARCA1* gene have divergent patterns of expression in cancers, such as colon adenocarcinoma (COAD), LUAD and stomach adenocarcinoma (STAD) (Fig. 2A), suggesting that they have differential roles or even opposing roles. Indeed, SMARCA5-null embryos die during the peri-implantation stage due to hypoproliferation of the inner cell mass and trophoectoderm, while SMARCA1-null mice survive normally, but show hyperproliferation of cortical progenitors, resulting in an enlarged brain [47]. Therefore, changing the balance of SMARCA5 and SMARCA1 levels could be a potential therapeutic strategy after confirming their opposing expression levels and functions in one given tumor.

### RSF complex

RSF1 functions as a nuclear protein with histone chaperon function and interacts with SMARCA5 or SMARCA1 to form RSF complexes that is involved in nucleosome assembly and ATPase-dependent chromatin remodeling [48] (Fig. 1A). The formation of RSF-5 complex is conducive to the nuclear import of SMARCA5, and stabilize SMARCA5 levels through preventing SMARCA5 protein degradation [49]. RSF1 is overexpressed in various tumor tissues based on recent studies and the TCGA database, including breast cancer, HCC, Glioma, etc. [50–55] (Fig. 2A). Meanwhile, overexpression of it is associated with poor overall survival, advanced clinical features and drug resistance in many types of cancer [50–57]. Mutations and abnormal copy numbers have also been found in a variety of tumors, such as acral melanoma, STAD, bladder cancer, etc. (Tables 2 and 3). As a binding partner, SMARCA5 is also overexpressed in the above-mentioned RSF1-overexpressing tumors, as reflected by recent reports [42, 50, 55] and the TCGA database analysis (Fig. 2A).

RSF1 was identified as an amplified gene that activated NF-κB–dependent gene expression such as *XIAP* (antiapoptotic gene) and *PTGS2* (anti-inflammatory gene) for paclitaxel resistance by functioning as a coactivator for the NF-κB-SMARCA5 complex in ovarian tumor cells [56] (Fig. 4B). In some case, the functional characteristics of RSF1 for malignant transformation are associated with p53 expression or its mutation status. For example, in non-transformed cells, upregulation of RSF1 induces an ATM/p53-dependent DNA damage response, which leads to growth arrest and apoptosis in cells with wild-type p53. Inactivation of p53 by knockout of p53 alleles or p53 mutation reverses the growth inhibitory effects of RSF1 and favors outgrowth of cell clones [48, 58]. In contrast, upregulation of an cyclin E1-RSF1-SMARCA5 complex promotes tumorigenicity in the presence of p53^mut^ RK3E cells, while tumorigenesis was not detected if they were expressed in a p53^wt^ background [59]. In addition, Sehdev et al. found that p53 mutations probably represent a very early molecular genetic change in the development of high-grade serous carcinoma, which initiates a cascade of molecular changes, including upregulation of RSF1 [60]. These studies suggest that RSF1 may act as a driver gene for tumorigenesis, confer on cells with a p53 mutation background certain selective advantages with respect to neighbouring cells.

### ACF complex

BAZ1A forms ACF complexes together with SMARCA1/5 or CHRAC complexes with SMARCA1/5, CHRAC1 and POLE3 (Fig. 1A). The ACF complex catalyzes both the relaxation of chromatin structure and the deposition of histones into extended periodic nucleosome arrays [61]. Within the ACF complex, BAZ1A stimulates SMARCA5 activity, regulates SMARCA5 remodeling properties and enhances the efficiency of nucleosome sliding during DNA replication and transcription, while SMARCA5 maintains the stability of the BAZ1A protein [11, 62]. Furthermore, CHRAC1 and POLE3 interaction facilitates nucleosome sliding and assembly mediated by the BAZ1A-CHRAC complex [63].

In normal cells, BAZ1A is involved in DNA double-strand breaks (DSBs) and DSB repair. Upon DNA damage, BAZ1A and SMARCA5 accumulate rapidly at DSBs and contribute to nonhomologous end-joining (NHEJ) repair of DSBs via recruitment of the KU70/80 complex and formation of the CHRAC complex. Inhibition of BAZ1A or SMARCA5 causes cells to become extremely sensitive to X-rays and chemical treatments with unrepaired DSBs [62]. The ACF complex is also associated with cellular senescence. Li et al. found that disruption of the ACF complex either by inhibition of BAZ1A or SMARCA5 resulted in the upregulation of the target gene *SMAD3*, which in turn activated *p21* gene transcription and senescence-associated phenotypes of tumor cells [64].

Through TCGA database analysis, we found that BAZ1A, CHRAC1 and POLE3 are simultaneously up-regulated in a variety of tumors, such as esophageal carcinoma (ESCA), liver hepatocellular carcinoma (LIHC), STAD, breast invasive carcinoma (BRCA), etc. (Fig. 2A). Specially, various kinds of tumors, such as adrenocortical carcinoma, gastric cancer, melanoma, etc. display high frequency of abnormal copy numbers in at least two components of CHRAC complexes (Table 3). These data suggest that CHRAC complexes potentially play a driving role in cancers. Indeed, studies found that the *BAZ1A* gene within the 14q12-q13 amplicon is frequently amplified in ESCC, while this region harbors some oncogenic genes [27]. *CHRAC1* gene, amplified on chromosome 8q24.3, is confirmed to be a driver gene regulating the proliferation/survival of clonogenic breast cancer cells [65]. In prostate cancer, *POLE3* is upregulated by aberrant copy number amplification in cisplatin-resistant testicular germ cell tumors harboring the 9q32-q33.1 gain [66]. Knockout of the *POLE3* gene increased the sensitivity of cells to an ATR inhibitor, a PARP inhibitor, and camptothecin [67].

### BAZ1B

BAZ1B, a tyrosine-protein kinase belonging to the bromodomain family, was originally identified as a hemizygously deleted gene in Williams syndrome [68]. BAZ1B interacts with SMARCA1/5 to form WICH complexes (Fig. 1A). BAZ1B binds specifically to acetylated histones and plays a critical role in chromatin assembly, RNA polymerase I and III gene regulation, vitamin D metabolism, and DNA repair. Acetylation or phosphorylation (S158) of BAZ1B increases intrinsic kinase activities [69]. BAZ1B is overexpressed in breast and colorectal carcinoma cancer tissues, as well as in ESCA, STAD, etc. from TCGA database (Fig. 2A). Moreover, BAZ1B possesses high frequency of mutation in cutaneous cancer and lung carcinoma as well as high frequency of copy numbers amplification in breast cancer, gastric cancer and etc. (Tables 2 and 3).

In breast cancer, BAZ1B acts as an activator of a *CYP19A1* gene that encodes the enzyme aromatase and *ERα* genes [70]. Treatment with the vitamin D analog EB1089 effectively inhibits aromatase-dependent growth of breast cancer cells by dissociating BAZ1B from the *CYP19A1* promoter in a vitamin D receptor (VDR)-dependent manner, suggesting that BAZ1B is a potential drug target in breast cancer [70, 71] (Fig. 4C). In lung cancer, Meng et al. found that BAZ1B acts as an oncogene to promote tumor aggressiveness by inducing epithelial–mesenchymal transition (EMT) via activation of the PI3K/Akt and IL-6/STAT3 pathways [72].

BAZ1B functions not only in intranuclear transcription regulation but also in intercellular communication. Liu et al. found that the KRAS^G12^ mutant induces the release of BAZ1B into the extracellular space by activating *NRG3* transcription that can transport BAZ1B [73]. A BAZ1B-NRG3 complex in the extracellular space activates a series of oncogenic pathways such as the RAS-MAPK, NOTCH1 and JAK pathways, in normal colon cells carrying KRAS^WT^ and endows them with the ability to express NRG3 and release BAZ1B-NRG3 complexes, thereby promoting the transformation of surrounding normal cells in a cascaded manner [73]. BAZ1B is also involved in drug response. Blockade of extracellular BAZ1B restores the cetuximab sensitivity of colon cancer cells with mutant KRAS [73, 74]. Moreover, BAZ1B silencing synergistically potentiates the anti-growth effects of bortezomib in myeloma [75]. Phosphorylation of histones by BAZ1B is a key epigenetic mechanism that mediates UV and drug responses. In breast, prostate and bone tumor cells, BAZ1B, INTS3 and RUNX2 form UV-responsive complexes with the serine-139-phosphorylated isoform of H2AX (γH2AX). This complex supports histone displacement, DNA unwinding and stabilization of single-stranded DNA to mount an integrated response to DNA damage [76]. Like BAZ1A, BAZ1B has high-frequency mutations in skin melanoma cells (Table 2), which provides an intriguing link to UV-sensitivity. Upregulation of BAZ1B by imatinib is involved in apoptosis of CML cells by phosphorylation of H2AX at Tyr142 [77]. Moreover, BAZ1B acts as a key mediator that connects Ras/ERK signaling and phosphorylation of H2AX (H2AX^Y142ph^). Ras-ERK1/2 induces BAZ1B degradation by increasing MDM2 expression to downregulate H2AX^Y142ph^, which promotes cell growth and metastasis in gastric cancer [78].

### NORC complex

NORC complexes, composed of BAZ2A and SMARCA1/5, recruit promoter-bound TTF-I, pRNA, and acetylated H4K16 to ribosomal DNA (rDNA) through BAZ2A’s TAM bromodomain domain [79, 80]. In human, rDNA instability is observed in cancers and premature aging syndromes. The NORC complex is critical for preventing cellular senescence through interaction with SIRT7, which maintains rDNA heterochromatin [81]. Depletion of BAZ2A unleashes rDNA instability, with excision and loss of rDNA gene copies, which in turn induced acute senescence [82].

BAZ2A are up-regulated in multiple tumors, such as prostate cancer, HCC, and chronic lymphocytic leukaemia (CLL) [28, 83, 84]. Moreover, BAZ2A are overexpressed in ESCA and STAD with high frequency of mutations according to TCGA database (Fig. 2A and Table 2). BAZ2A has been found to act as an oncogenic partner of TFs [85–87]. Upregulation of BAZ2A in HCC promoted the EMT progression of HCC cells by enhancing the interaction between β-catenin and TCF7L2 through its interaction with TCF7L2 [83]. Moreover, a fusion of ETV6 with the BAZ2A intron sequence generated by a cytogenetically cryptic rearrangement between 12p13 and 12q13 occurred in a pediatric case of pre-B acute lymphoblastic leukemia (ALL), which encodes a truncated form of ETV6 that leads to a pathogenic effect [88]. In a mouse prostate cancer organoid model, high BAZ2A expression is critical for the initiation of prostate cancer of luminal origin mediated by Pten-loss whereas it is dispensable once Pten-loss mediated transformation is established, which suggest that BAZ2A-mediated epigenetic second events sensitize for key genetic events that drive tumor development [89]. Mechanismly, BAZ2A binds to a class of inactive enhancers that are marked by H3K14ac via its bromodomain and represses the expression of genes implicated in aggressive and dedifferentiated prostate cancer [90].

### CERF complex

The CERF complex remodels chromatin via nucleosome-dependent ATPase activity and is involved in DNA double strand break repair [91, 92]. Recently, CECR2 together with nine other genes, including *ARHGAP21, ENSA, GPATCH8, KIAA1109, MGMT, PCDHB13, SELM, SPAG9* and *WDR6*, have been reported to be selected as the optimal gene combination that could be associated with the prognosis of patients with glioma [93]. Furthermore, a non-BET BRD inhibitor, NVS-CECR2–1, is able to kill SW48 colon tumor cells by targeting CECR2, suggesting that it may be an oncogene in some types of tumors [94]. However, the molecular and clinical implications of CECR2 in cancer remain largely unknown.

### NURF complex

The NURF complex catalyzes ATP-dependent nucleosome sliding. Within the complex, BPTF, recognizes histone loci of methylation (H3K4me2/3) and acetylation (H4K12/16/20) by its PHD finger domain and bromodomain, respectively, and thus promotes gene transcription [31]. RBBP4 and RBBP7 are members of the WD-40 protein family and originally found associated with retinoblastoma proteins [95]. Both proteins are components of several multi-protein complexes that contain histone deacetylases (HDACs) and are involved in chromatin remodeling, histone post-translational modifications and regulation of gene expression, suggesting diverse functions for RBBP4/7 [95, 96].

### BPTF

Genetic disorders of the *BPTF* gene are commonly seen in various cancer tumors. According to TCGA database, BPTF is upregulated in STAD, ESCA, LIHC, LUAD and downregulated mainly in kidney Chromophobe (KICH), thyroid carcinoma (THCA), kidney renal clear cell carcinoma (KIRC), prostate adenocarcinoma (PRAD), etc. (Fig. 2A). Moreover, BPTF possess both high frequency of mutation and abnormal copy number in angiosarcoma, COAD, ESCA, LIHC, LUAD, melanoma and urothelial carcinoma (Tables 2 and 3) [23, 24]. Particularly, *BPTF* was found to be the most frequently mutated gene observed (28.6%) in whole exome sequencing of Lacrimal Gland Adenoid Cystic Carcinoma (LGACC) [97]. Moreover, a NUP98-BPTF gene fusion in which a C-terminal chromatin recognition module of BPTF is fused to the N-terminal moiety of NUP98 has been identified in primary refractory acute megakaryoblastic leukemia (AMKL) that contributes to refining the NUP98 rearrangement subgroup of pediatric AMKL [98].

BPTF functions as a cofactor of oncogenic TFs. For example, BPTF-NURF complex is essential for c-MYC recruitment, chromatin accessibility and remodeling [99–101] (Fig. 3C). Knockdown of BPTF impairs tumor development with inactivation of the *c-MYC* and/or c-Myc target genes transcriptional program in preneoplastic pancreatic acinar cells and high-grade glioma [99, 101]. Correspondingly, BPTF expression is positively correlated with *c-MYC* gene signatures [99–101]. Particularly, the epigenetic environment is important for the genomic locations of BPTF. In bladder cancer, H2A.Z nucleosomes are enriched for the active histone modification H3K4me2/me3, which facilitates to recruit BPTF to H2A.Z target genes [102, 103]. The BPTF-c-Myc signaling axis is suggested to be a driving factor. For example, In the Eμ-Myc transgenic mouse model of aggressive B-cell lymphoma, BPTF allele is sufficient to delay lymphomagenesis, which display decreased c-MYC levels and pathway activity [104], suggesting that interruption of the c-Myc-BPTF-NURF complex interaction is a potential strategy for the treatment of c-Myc driven tumors.

### RBBP7

Through the TCGA database analysis, RBBP4/7 and BPTF is simultaneously overexpressed in ESCA, LIHC, STAD etc. and downregulated in KIRC, KICH, kidney renal papillary cell carcinoma (KIRP) and prostate adenocarcinoma (PRAD) (Fig. 2A). RBBP4/7 expression were also consistent with HCC severity and prognosis together with BPTF [39]. Moreover, RBBP4 or RBBP7 and BPTF possess high frequency of abnormal copy number in angiosarcoma, breast cancer, pancreatic cancer, prostate cancer and gastric cancer (Table 3), and are mutated in colorectal adenocarcinoma, lymphoma, melanoma, etc. (Table 2).

**Fig. 5:**
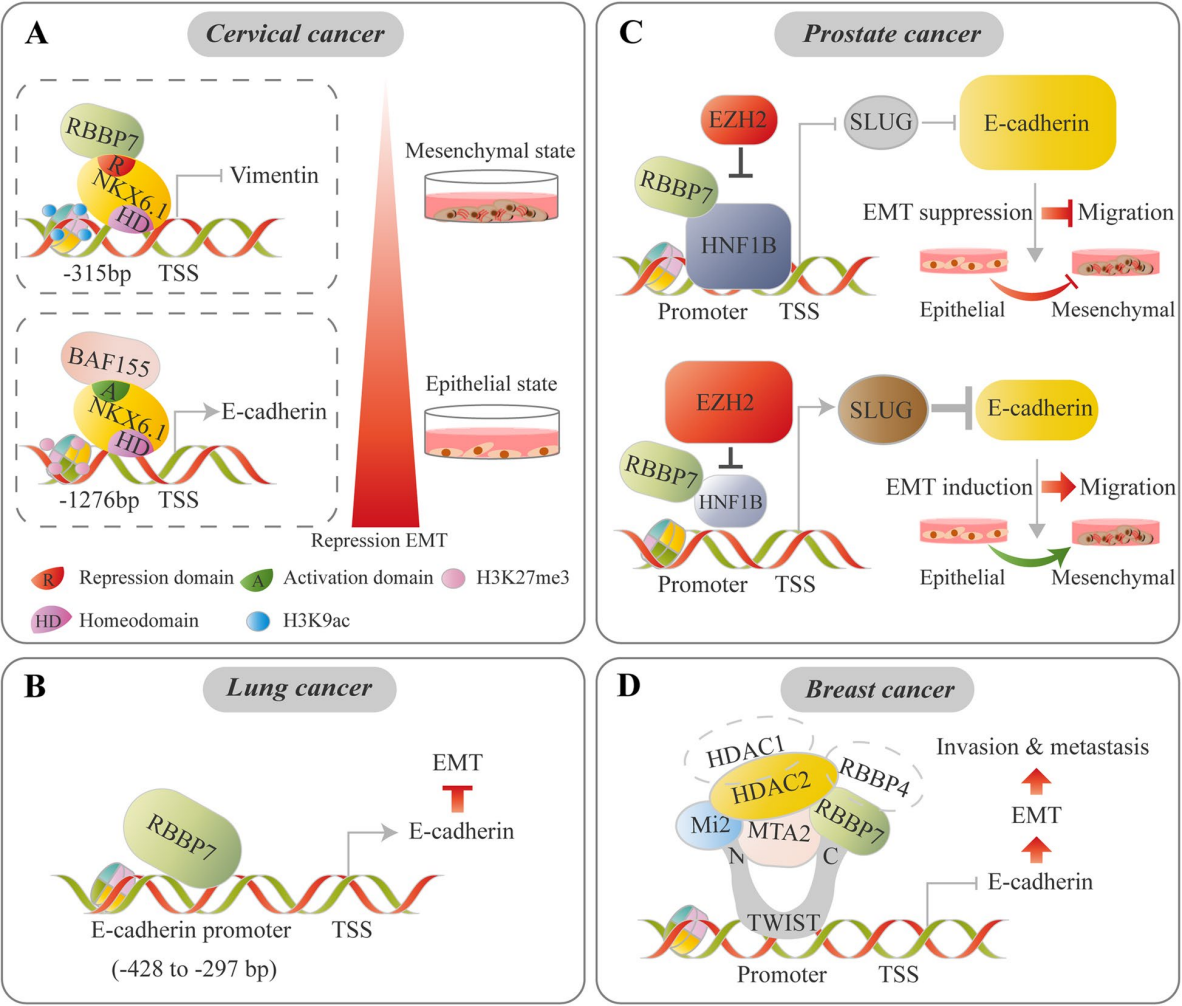
ISWI-mediated EMT regulation is critical for tumor progression. ISWI proteins function as cofactors together with specific TFs to modulate EMT-related genes in a context-dependent manner. **A** In cervical cancer, NKX6.1 directly represses *vimentin* by interacting with the RBBP7 corepressor, accompanied by an increased H3K27me3 level. Meanwhile, NKX6.1 directly activates *E-cadherin* by interacting with the BAF155 coactivator with an increased H3K9 acetylation level [105]. **B** In lung cancer cells, RBBP7 acts as a transcriptional activator of the *E-cadherin* gene by binding to its promoter region, thereby repressing EMT progression [106]. **C** Normally, RBBP7, as a corepressor, interacts with HNF1B to repress *SLUG* transcription and EMT progression. In prostate cancer, upregulation of EZH2 suppresses the levels of the RBBP7/HNF1B transcriptional complex via direct inhibition of HNF1B expression, promoting SLUG transcription and EMT progression [107]. **D** RBBP7, as a corepressor, suppresses *E-cadherin* by interacting with TWIST and recruiting the complex to proximal regions of the *E-cadherin* promoter, thus inducing EMT [108]

A dual role of RBBP7 in tumor metastasis has been found to be closely related to recruitment of different RBBP7-containing complexes to EMT-related genes. For example, in cervical cancer, RBBP7 and BAF155 can be differentially recruited by NKX6.1 to suppress the *vimentin* gene and activate the *E-cadherin* gene, thus suppressing the EMT program [105] (Fig. 5A). In lung cancer, the region between − 428 and − 377 of the E-cadherin promoter contains a binding site for RBBP7, where RBBP7 functions as a transcriptional activator of the *E-cadherin* gene by binding to its promoter region, thereby repressing EMT progression [106] (Fig. 5B). Furthermore, RBBP7, as a corepressor, interacts normally with HNF1B to repress the *SLUG* gene and EMT program. However, in prostate cancer, upregulation of EZH2 suppresses the levels of the RBBP7/HNF1B transcriptional complex via direct inhibition of HNF1B expression, promoting *SLUG* transcription [107] (Fig. 5C). Additionally, in breast cancer, the transcription factor TWIST recruits an RBBP7/4-MTA2/Mi-2/HDAC1/HDAC2 complex to the proximal regions of the *E-cadherin* promoter for transcriptional repression via an interaction with RBBP7, which promotes breast cancer cell invasion and metastasis [108] (Fig. 5D).

### RBBP4

RBBP4-containing complexes are involved in drug resistance. RBBP4 interacts with CBP/p300 to form a chromatin modifying complex that binds to MGMT, RAD51 and selected DNA repair genes. RBBP4 deletion enhanced temozolomide (TMZ) sensitivity, induced synthetic lethality to PARP inhibition and increased DNA damage signaling in response to TMZ in glioblastoma [109]. Release of RBBP4 complex occupancy or targeting the interactions between RBBP4 and oncogenic TFs provides increased opportunities for tumor intervention. For example, a BCL11A peptide inhibitor that blocks the recruitment of RBBP4-BCL11A complexes to BCL11A-targeted genes decreases aldehyde dehydrogenase-positive breast cancer stem cells (BCSCs) [110]. FFW functions as a specific SALL4–RBBP4 inhibitory peptide by targeting RBBP4 in HCC, releasing the expression of tumor suppressor PTEN [111] (Fig. 4D). In neuroblastoma cells, RBBP4 interacts with ARMC12 to facilitate the enrichment of PRC2 and H3K27me3 on tumor suppressive genes such as *CADM1*, *EGLN3* and *SMAD9*, resulting in transcriptional repression. While a cell-penetrating inhibitory peptide blocks the interaction between ARMC12 and RBBP4, inhibiting aggressive cell behaviors [112].

However, although some ISWI dependent functions of RBBP4/7 as components of NURF complexes have been uncovered [38, 39, 113–118]. It should be noted that RBBP4 and RBBP7 not only participate in ISWI complexes, but also in several multi-protein transcription complexes [95, 110, 119]. Specially, ISWI participates in different larger protein complexes [120–122]. At present, a large proportion of studies mentioned above focus on the biological functions and/or mechanisms of RBBP4 and RBBP7 alone, which can not make a conclusion that RBBP4 or RBBP7 play those roles in some given tumors in a ISWI or non-ISWI dependent manners. It is possible that both ISWI or non-ISWI dependent mechanisms simultaneously contribute to the whole impact of RBBP4 or RBBP7.

### BRF complexes

BAZ2B interacts with SMARCA1/5 to form BRF-1 and BRF-5 complexes, which induces remodeling of the DNA-bound mononucleosomes [22]. Crystal structure studies of purified BAZ2B protein show that its PHD domain interacts with unmodified histone H3K4 while the bromodomain interacts with the acetylated histone marks on H3K14 and H3K16 [123]. According to the TCGA database, *BAZ2B* is down-regulated in a variety of tumors, such as KICH, lung squamous cell carcinoma (LUSC), BRCA, etc. (Fig. 2A) Moreover, BAZ2B possess highly frequency mutations and abnormal copy number in some tumors, such as melanoma, bladder cancer, COAD, HCC, etc. (Table 2 and Table 3) However, its pathogenic mechanisms still remain unclear, and need to be further explored.

### The ISWI and non-coding RNA functional connection

**Fig. 6:**
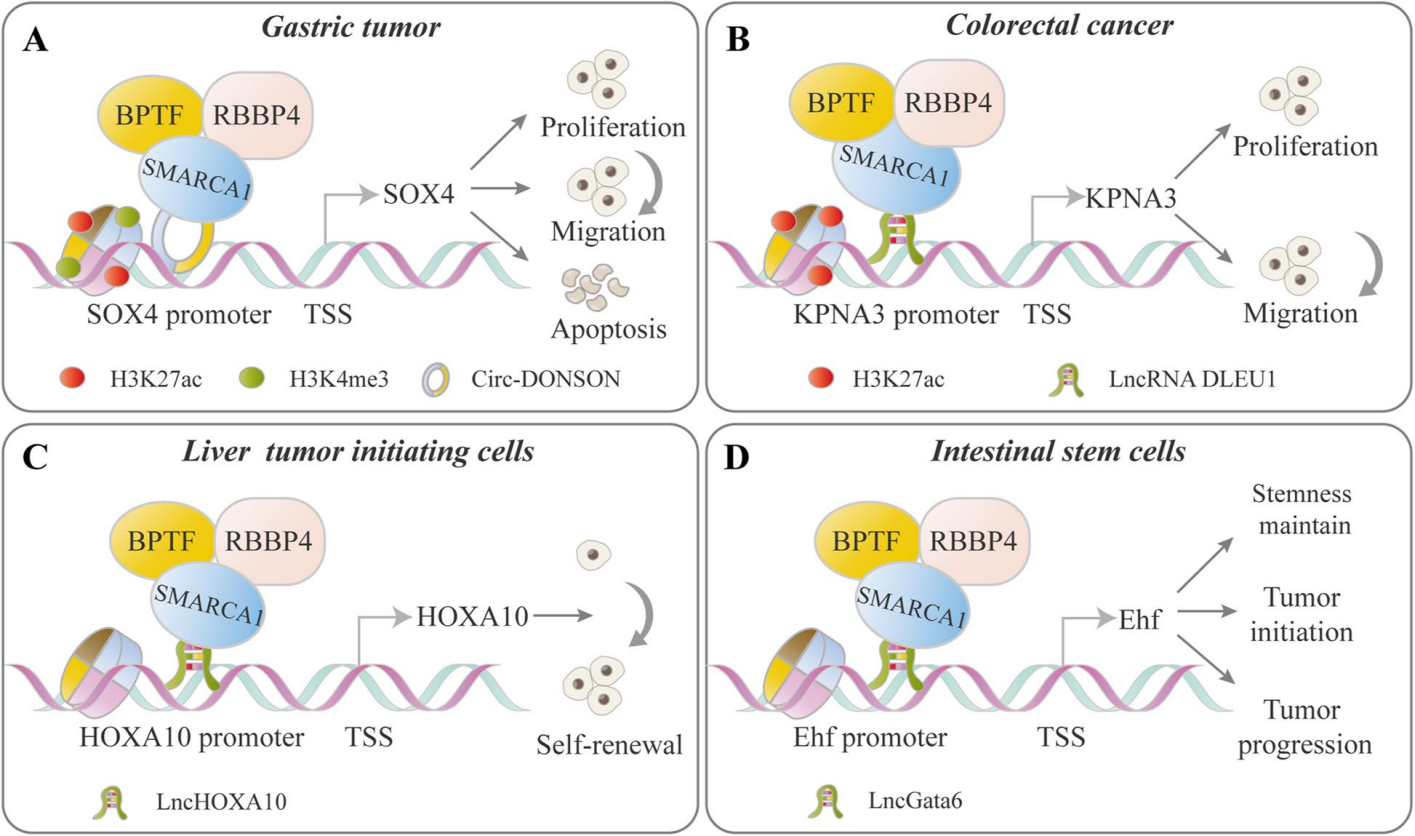
Representative models of ISWI-noncoding RNA interplay in cancer. ISWI family proteins are implicated in the regulation of oncogene transcription involving their interplay with noncoding RNAs. Generally, noncoding RNA recruits ISWI proteins to alter the chromatin environment and histone modification, thereby affecting oncogene transcription. **A** In gastric tumors, circ-DONSON recruits the SNF2 L-NURF complex to the *SOX4* promoter by interacting with SMARCA1, which enriches the transcriptionally active markers H3K27ac and H3K4me3 and increases *SOX4* promoter accessibility and transcription [115]. **B** In colorectal cancer, lncRNA DLEU1 recruits the SNF2 L-NURF complex to the *KPNA3* promoter and promotes gene expression through the enrichment of histone modification H3K27ac, which further facilitates malignant behaviors [116]. **C** LncHOXA10 guides the localization of the SNF2 L-NURF complex to the *HOXA10* promoter by directly interacting with SMARCA1, which further activates *HOXA10* transcription and promotes liver tumor-initiating cell self-renewal [117]. **D** lncGata6 recruits the NURF complex to the *Ehf* promoter and induces gene expression by directly binding with SMARCA1, which further promotes Lgr4/5 (ISC cell-specific marker) expression and activation of Wnt signaling and thus maintains ISC stemness or promotes tumorigenesis [118]

Recent studies showed that non-coding RNAs regulate chromatin modification and gene expression through their interactions with the ISWI complexes, which affect tumorigenesis and development in different ways. Non-coding RNAs can directly bind to the subunit of the ISWI complexes and serve as a guide to anchor the ISWI complexes. Moreover, Non-coding RNAs can be incorporated into the ISWI complexes and function as a scaffold to assemble the complex for chromatin remodeling. For example, in gastric tumor cells, the circ-DONSON recruits the SMARCA1-NURF complex to the *SOX4* promoter by directly interacting with SMARCA1, which facilitates tumor cell development via enrichment of the active markers H3K27ac and H3K4me3 on the promoter and activation of *SOX4* transcription [115] (Fig. 6A). In colorectal cancer, the SMARCA1-NURF complex is recruited by lnc-DLEU1 to the *KPNA3* promoter and initiates *KPNA3* expression via H3K27ac enrichment, which promotes tumor cell proliferation and migration [116] (Fig. 6B). SMARCA1-noncoding RNA complexes also play a vital role in tumor-initiating cells (TICs) and their properties. lncHOXA10 recruits the SMARCA1-NURF complex to the *HOXA10* promoter and activates gene transcription, promoting the self-renewal of liver TICs [117] (Fig. 6C). Similarly, lncGata6 guides the localization of the SMARCA1-NURF complex to the *Ehf* promoter, which subsequently induces *Lgr4/5* expression and activation of Wnt signaling in intestinal stem cells (ISCs) [118] (Fig. 6D). ISWI proteins are also involved in the noncoding RNA regulatory network. For example, RSF1 functions as an effector in lncRNA-induced drug resistance. In NPC, NEAT1/let-7a-5p axis regulates the cisplatin resistance by targeting RSF1 [124]. In ESCC, NSUN2-methylated lncRNA (NMR) directly bind to BPTF and potentially elevate MMP3 and MMP10 expression by the ERK1/2 pathway by recruiting BPTF to chromatin [125].

In particular, recent studies found that circRNAs transcribed from the ISWI genes were involved in the etiology of cancer. cSMARCA5, a circRNA derived from exons 15 and 16 of the *SMARCA5* gene (hsa_circ_0001445) was upregulated in prostate cancer. Knockdown of cSMARCA5 significantly repressed the cell cycle and promoting tumor apoptosis [126]. The presence of cSMARCA5 inhibits the growth and metastasis of HCC by acting as a sponge of miR-17-3p and miR-181b-5p to upregulate TIMP3 [127, 128]. Interestingly, cSMARCA5 and SMARCA5 displayed opposite expression in HCC. Both products of the *SMARCA5* gene and cSMARCA5 are associated with poor prognosis, synergistically promoting the progression of HCC [43, 127, 128]. Circ-BPTF also plays an important role in tumor progression. For example, a circular RNA (hsa_circ_0000799) derived from BPTF exons attenuates the anti-oncogenic effect of miR-31-5p and consequently enhances RAB27A expression in bladder cancer [129].

### Impact of ISWI proteins on tumor immunity

**Fig. 7:**
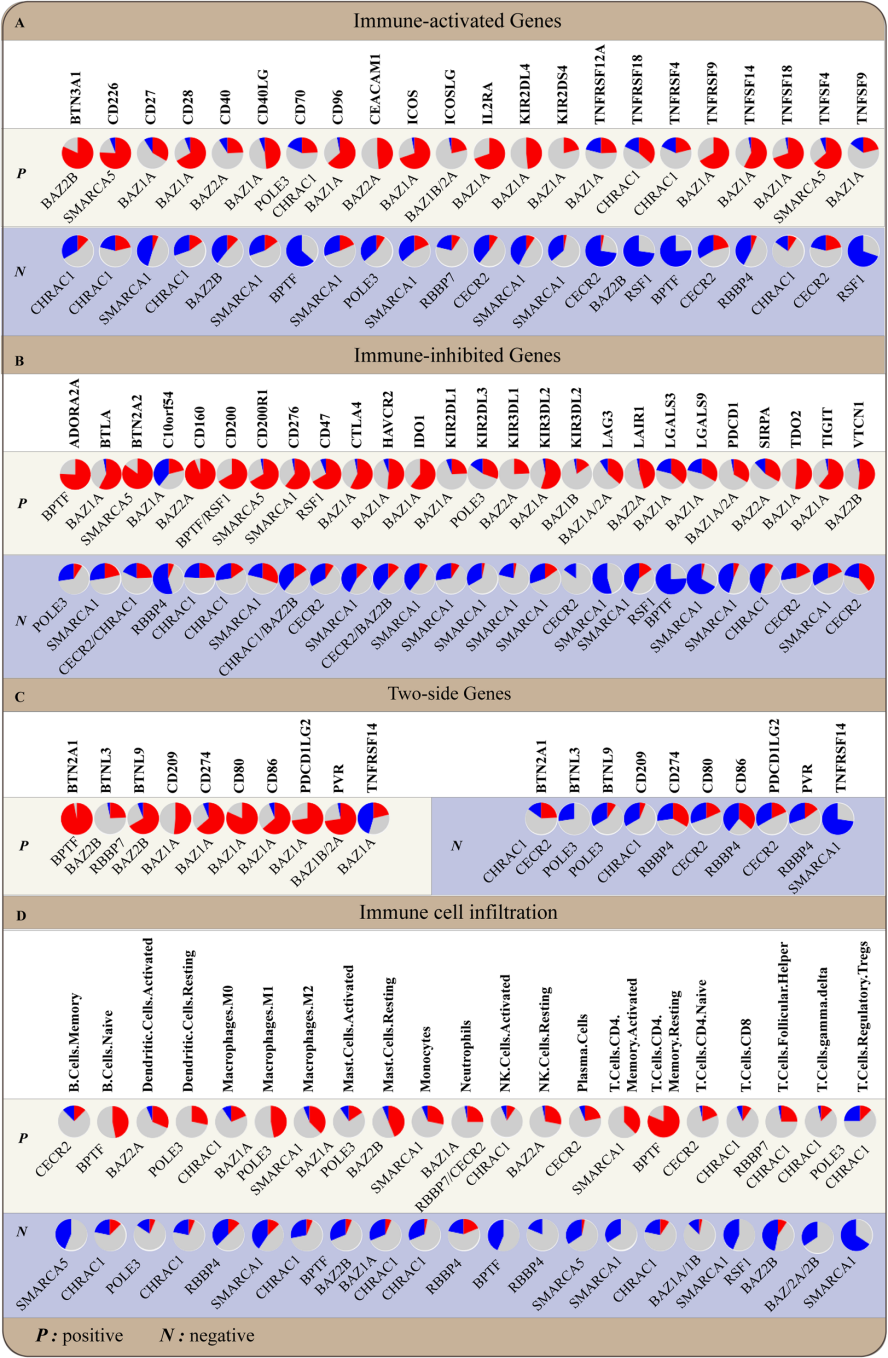
The mRNA expression levels of ISWI members correlate with immune checkpoint gene levels and immune cell infiltration. The most significant correlation between ISWI members and immune checkpoints at the gene level or between the gene level of ISWI and immune cell infiltration in the 33 tumors from TCGA database are shown in the pie chart. Spearman’s method was used to calculate the correlation between ISWI family members and immune checkpoints (**A-C**) or immune cell infiltration (**D**) in 33 tumors. The CIBERSORT algorithm was used to calculate the proportion of different types of cells. Red indicates a positive correlation, blue indicates a negative correlation, and gray indicates no significant correlation. Cancer is abbreviated as follows: Esophageal carcinoma (ESCA), Liver hepatocellular carcinoma (LIHC), Rectum adenocarcinoma (READ), Colon adenocarcinoma (COAD), Lung adenocarcinoma (LUAD), Stomach adenocarcinoma (STAD), Head and Neck squamous cell carcinoma (HNSC), Bladder Urothelial Carcinoma (BLCA), Breast invasive carcinoma (BRCA), Lung squamous cell carcinoma (LUSC), Uterine Corpus Endometrial Carcinoma (UCEC), Kidney renal clear cell carcinoma (KIRC), Thyroid carcinoma (THCA), Kidney Chromophobe (KICH), Kidney renal papillary cell carcinoma (KIRP), Prostate adenocarcinoma (PRAD)

The levels of tumor-infiltrating immune cells within the tumor microenvironment (TME) and immune checkpoints on immune cells and tumor cells are two key factors determining the antitumor immune response. A correlation analysis in 33 tumor types based on the TCGA database showed that the gene levels of some ISWI members are strongly correlated with immune checkpoint gene levels and/or the tumor-infiltrating immune cell ratio within the TME, suggesting that ISWI members play a key role in the transcriptional regulation of immune-related genes and pathways (Fig. 7). To date, BPTF, POLE3, RSF1, BAZ1B and CECR2 have been found to be involved in immune cell development and activity and the regulation of immune-related genes.

The BPTF-NURF complex plays vital roles in normal thymocyte development. This complex facilitates the differentiation of CD4+ and CD8+ cells to mature T cells by activating the transcription of key thymocyte maturation-specific genes (e.g., *Egr1, Ikaros, IL-2,* and *IL7ra*) in a T-cell antigen receptor signaling-dependent manner [130]. For example, BPTF facilitates the recruitment of the NURF complex to the promoter region of the *Egr1* gene via interaction with TF SRF [2, 130]. Aberrantly high levels of the BPTF/NURF complex are supposed to promote cancer immune escape by impacting multiple types of immune cell activity. Deletion of BPTF activates a stimulatory molecule and inhibits an inhibitory antigen on the surface of mouse breast cancer and skin melanoma cells, inducing a T-cell mediated immune response [131]. In BPTF knockout mouse models of breast cancer and melanoma, BPTF depletion enhances antigen processing and CD8+ T cell cytotoxicity [131]. Mechanistically, BPTF knockout promotes the expression of the immunoproteasome subunits Psmb8 and Psmb9 and the antigen transporters Tap1 and Tap2, resulting in enhanced antigenicity and T-cell antitumor immunity [131]. Tumor cells with BPTF deficiency display increased CD8+ cell infiltration and CD8+ cell cytotoxicity, including the release of perforin, granzyme and IFN-γ and subsequent induction of the JAK/STAT and Fas/TRAIL pathways [132]. BPTF also stimulates heparanase expression, which reduces cell surface heparan sulfate proteoglycan and natural cytotoxicity receptor co-ligand abundance, therefore inhibiting NK cell antitumor activity in breast cancer cell lines [33]. Moreover, BPTF is vital for self-tolerance and immune homeostasis by stabilizing Treg function and Foxp3 expression in a cell-intrinsic manner [133]. Lack of BPTF in Foxp3-expressing Treg cells caused defective suppressive function of Treg cells, reduced Foxp3 expression, and increased lymphocyte infiltration in the nonlymphoid organs, ultimately leading to aberrant immune activation and an autoimmune syndrome [133].

In addition to BPTF, POLE3, RSF1 and BAZ1B also play vital roles in T cell-mediated immunity via regulation of cell cycle progression, antigen-specific cytotoxic T lymphocyte (CTL) responses and metabolic pathways. C-terminal mutants of POLE3 cause a cell-autonomous, stage-specific interruption of T and B cell development and interfere with the S-phase of cell cycle progression in lymphocytes, leading to severe peripheral lymphopenia [134]. RSF1-transduced dendritic cells induced a CTL response to produce IFN-γ and IL-12 against ovarian cancer cells in vitro, suggesting that RSF1-transduced dendritic cells may be a potential adjuvant immunotherapy [135]. BAZ1B functions as an amino acid sensor to modulate T cell antitumor immunity, cytokine production and survival [136]. Upregulated L-arginine levels have been reported to induce global metabolic changes, including a conversion from glycolysis to oxidative phosphorylation in activated T cells, and increase the generation of central memory-like cells with higher survival capacity and antitumor activity [136]. Interestingly, BAZ1B, PSIP1, and TSN could sense and modulate the L-arginine-dependent reprogramming of T cells toward increased survival capacity during the above process [136–138].

CECR2 has been found to be involved in the regulation of tumor immunity via macrophages. In this respect, upregulation of CECR2 in metastatic breast cancer is positively related to M2 macrophages and increases tumor metastasis by promoting M2 macrophage polarization to create an immunosuppressive microenvironment [139]. Mechanistically, CECR2 formed a complex with p65 through its bromodomain to activate the expression of the NF-κB target genes *CSF1* and *CXCL1*, which are critical for macrophage-mediated immune suppression at metastatic sites [139]. Correspondingly, the inhibition of CECR2 by targeting bromodomain arrests immunosuppression by macrophages and inhibits breast cancer metastasis [139].

### ISWI complexes as potential targets for cancer therapy

The emergence of ISWI members as oncology targets has spurred significant drug discovery efforts with the goal of identifying small molecule inhibitors that target their functional domains for therapeutic applications [140]. The bromodomain of ISWI is an attractive target for drug design. Bromodomains are readers of acetyl marks in histone tails or nonhistone proteins. From the perspective of bromodomain structure, the bromodomain structure possesses a hydrophobic acetylated lysine-binding pocket, which is optimal for the interaction of the charge-neutralized acetylated lysine and has comparatively low strength of protein-protein interaction, thus making this domain particularly targetable by small molecules that interfere with this interaction [141]. Several potent inhibitors are being developed for bromodomains present in the ISWI complexes. NVS-CECR2–1, the first selective inhibitor targeting the CECR2 bromodomain, inhibits chromatin binding of CECR2 bromodomain and displaces CECR2 from chromatin within cells. NVS-CECR2–1 exhibits cytotoxic activity against various human cancer cells mainly through inducing cell apoptosis [94]. GSK2801 is a selective and cell-active acetyl-lysine competitive inhibitor of BAZ2A/B bromodomains. Although GSK2801 has little effect on growth arrest as a single agent, it shows a strong synergistic effect on triple-negative breast cancer (TNBC) in combination with the BET bromodomain inhibitor (BETi) JQ1 [142]. Their synergistic inhibition on bromodomains induces apoptosis of TNBC by a combinatorial suppression of ribosomal DNA transcription and ETS-regulated genes. Arylurea (AU1) was the first small molecule selective inhibitor of the BPTF bromodomain and was selective for BPTF over BRD4 with moderate potency in an in vitro assay. AU1 treatment alters chromatin accessibility, decreases target gene c-MYC chromatin occupancy, weakens proliferative capacity, and leads to G1 arrest in mouse breast cancer cells [143].

The availability of crystal structures of ISWI subunits provided a unique strategy to develop their selective antagonists. To date, the crystal structures of ISWI subunits in some species have been elucidated to different resolution [144–147]. For example, Yan et al. crystalized a construct of ISWI containing the catalytic core from *M. thermophila* called MtISWI with 2.4 Å resolution, whose sequence is about 68, 68, and 58% identical to those of ISW1 and ISW2 of *Saccharomyces cerevisiae* and human SNF2h, respectively [148]. Besides, Chittori et al. determined a cryo-EM structure of the complex formed between nucleosome and the ATPase domain of the Chaetomium thermophilum ISWI [149]. In human, Armache et al. presented cryo-EM structures of the full-length form of the human ISWI remodeler at 3.4 Å, showing structures of SNF2h-nucleosome complexes with ADP-BeFx [150]. Tallant C et al. identified the high-resolution crystal structures of PHD zinc finger and BRD from of human BAZ2A and BAZ2B in complex with H3 and/or H4 histones from 1.6 Å to 1.99 Å [79].

## Conclusions and perspectives

As key chromatin remodeling complexes, ISWI variably functions as a part of different larger protein complexes, and different kinds of tumor cells have diverse expression arrays of ISWI components, each with discrete functions. Different ISWI subunits have unique regulatory roles and determine ISWI complex functions, which confer a complex ability to regulate a variety of cellular events in normal and malignant cells (Table 5). ISWI actions in cancer are gene- or context-dependent, while cooperation with different TFs or TCs may produce distinct tumor properties. The abnormal activity and expression of ISWI subunits or the occurrence of aberrant composition in ISWI-containing complexes could lead to malignant phenotypes by upsetting gene regulatory networks. In some cases, the function of oncogenic TFs and fusion proteins is reliant on direct interactions with ISWI-containing complexes, where the ISWI family is critical for the optimal oncogenic activity of the complexes. Many factors, such as mutation, copy number variations (CNVs) or aneuploidy, induce imbalances in ISWI subunit stoichiometry in cancers. In general, too few of any one subunit limits the number of assembled complexes that can satisfy biological functions. Conversely, excess subunits outside their designated complexes are often nonfunctional and may have adverse effects. Therefore, stoichiometric imbalances of key “driver” components of ISWI complexes in a given tumor may be a key etiology. Balancing or restoring the normal expression levels and/or function of components within ISWI complex or between ISWI and other protein complexes promises exciting therapeutic insights. ISWI bromodomain inhibitors have shown synergistic or additive effects with numerous chemotherapeutic agents. In preclinical and clinical settings. ISWI subunits have recently emerged as immunoregulators that modulate immune cell phenotypes or the expression of immune checkpoints. Therefore, the combination of ISWI inhibitors with immune checkpoint inhibitors may be considered a major breakthrough in the treatment of malignancies.

**Table 5:** ISWI-contaning complexes in cancer and their functions

Complex components	Cell types or tumors	Functions
SNF2L-NURF complex-Circ-DONSON	GC cell lines (BGC-823, AGS, MGC-803, MKN74, HGC-27 and SGC-7901)	Activating *SOX4* transcription, which leads to the proliferation, migration and invasion of GC cells
SNF2L-NURF complex-LncRNA DLEU1	Human colorectal cell lines (HCT8 and SW480 cell)	Initiating *KPNA3* expression and promoting cell proliferation, migration and invasion
SNF2L-NURF complex-LncHOXA10	Primary liver TICs	Activating *HOXA10* transcription to promote the self-renewal of liver tumor initiating cells (TICs)
SNF2L-NURF complex-LncGata6	Mouse intestinal stem cells	Promoting *Ehf* transcription, which subsequently induces Lgr4/5 expression and activation of Wnt signaling in intestinal stem cells (ISCs)
SNF2L-NURF complex-WASH	Mouse long term-hematopoietic stem cells	WASH assists the NURF complex in the c-Myc promoter and enhances gene transcription, which maintains the differentiation potential of long-term hematopoietic stem cells (LT-HSCs) to mature blood lineages
SNF2L-NURF complex-ZIC2	Primary liver CSCs and Hep3B cell lines	Initiating *OCT4* activation to maintain the self-renewal of liver CSCs
SNF2H-CTCF complex	MEL and OCI-M2 cells	Being recruited to the enhancer of *PU.1* gene and block gene expression
RSF1-cyclin E1	Renal epithelial cells	Contributing to neoplastic transformation in the presence of TP53 mutations
SNF2H-RSF1-NF-κB	Ovarian cancer cells lines (SKOV3, OVCAR3, and A2780)	Activating NF-κB–dependent gene expression, contributing to the development of chemoresistance
SNF2H-ACF1-CHRAC15-CHRAC17-KU70/80	U2OS/TRE/I-SceI-19 cells	Playing important roles on double-strand breaks repair
WSTF-RUVBL2- INTS3- RUNX2	Breast, prostate, and bone cancer cells lines (Saos2, U2OS, MDA-MB-231 and PC3)	Mounting an integrated response to DNA damage through supporting histone displacement, DNA unwinding, and stabilization of single-stranded DNA
WSTF-NRG3	Colon cancer cells line (SW48)	Activating oncogenic pathways of the surrounding normal colon cells through mediating cell–cell communication
TIP5-EZH2	Prostate cancer cell line (PC3)	Participating in epigenetic silencing of *AOX1*, *FBN1*, *FHL2* and *HOMER2* genes
BPTF-c-Myc	Pre-neoplastic pancreatic acinar cells	Increasing c-MYC recruitment to target genes and regulating chromatin accessibility at promoters, thus increasing target genes’ transcription
BPTF-P50-NF-κB	Lung cancer cell lines (A549 and NCI-H460)	Increasing *COX-2* expression by binding to *COX-2* promoter region, promoting tumor cell growth
BPTF-WDR5	Bladder cancer cell line (LD611)	Promoting the expression of tumorigenic genes
RBBP7-NKX6.1	Cervical cancer cell line (HeLa)	Serving as a repressor to bind to *vimentin* promoter, thus suppressing its transcription
RBBP7-HNF1B	Prostate cancer cell line (DU145)	Repressing *SLUG* expression and EMT phenotype
RBBP7/4-MTA2/Mi-2/HDAC1/2 -TWIST	Human and mouse breast cancer cells (MDA-MB-435 and 4 T1)	Suppressing *E-cadherin* transcription, inducing EMT program
RBBP4–BCL11A	Breast cancer cell line (SUM149)	The recruitment of RBBP4-BCL11A complexes to BCL11A-targeted genes decreases aldehyde dehydrogenase-positive breast cancer stem cells (BCSCs) and their mammosphere formation capacity
RBBP4-ARMC12	Neuroblastoma cell lines (SH-SY5Y, BE(2)-C and IMR32)	Facilitating the formation of PRC2 in neuroblastoma, resulting in transcriptional repression of tumor suppressive genes
RBBP4-CBP/p300	Glioblastoma cell line (T98)	Promoting DNA repair genes expression, which influences the survival against temozolomide (TMZ)therapy
RBBP4-LncRNA LCPAT1	Breast cancer cell lines (MCF-7 and MDA-MB-231)	Activating *MFAP2* transcription and promoting breast cancer progression

Given that epigenetic regulation of the ISWI family globally affects the gene regulatory network, an ensemble of key cancer-driving ISWI subunits has been identified. Future studies need to identify more regulatory factors that dysregulate ISWI family expression and determine how their misexpression contributes to the pathogenesis of malignancies. Identification of more crystal structures of ISWI subunits and epigenetic mechanisms, thereby targeting specific ISWI domains, ISWI-mediated pathways and the TF/ISWI interface, may contribute to the development of novel inhibitors in ISWI-associated malignancies.

## Data Availability

Not applicable.

## Abbreviations

AZA: 5-azacitidine
ALL: Acute lymphoblastic leukemia
AMKL: Acute megakaryoblastic leukemia
AML: Acute myeloid leukemia
AU1: Arylurea
BETi: BET bromodomain inhibitors
BRCA: Breast invasive carcinoma
BRD: Bromodomain
CSCs: Cancer stem cells
CRCs: Chromatin remodeling complexes
CHD: Chromodomain-helicase DNA-binding protein
CLL: Chronic lymphocytic leukaemia
COAD: Colon adenocarcinoma
CTL: Cytotoxic T lymphocytes
DSBs: Double-strand breaks
EMT: Epithelial–mesenchymal transition
ESCA: Esophageal carcinoma
ESCC: Esophageal squamous cell carcinoma
HCC: Hepatocellular Carcinoma
ISWI: Imitation switch
INO80: Inositol-requiring mutant 80
ISCs: Intestinal stem cells
KICH: Kidney Chromophobe
KIRC: Kidney renal clear cell carcinoma
KIRP: Kidney renal papillary cell carcinoma
LGACC: Lacrimal gland adenoid cystic carcinoma
LIHC: Liver hepatocellular carcinoma
LT-HSCs: Long term-hematopoietic stem cells
LADC: Lung adenocarcinoma
LUAD: Lung adenocarcinoma
LUSC: Lung squamous cell carcinoma
MFS: Metastasis-free survival
NPC: Nasopharyngeal carcinoma
NRG3: Neuregulin 3
NHEJ: Nonhomologous end-joining
NSCLC: Non-small cell lung cancer
OS: Overall survival
PDAC: Pancreatic ductal adenocarcinoma
PRAD: Prostate adenocarcinoma
RFS: Relapse free survival
RCC: Renal cell carcinoma
SCLC: Small cell lung cancer
STAD: Stomach adenocarcinoma
SWI/SNF: Switch/sucrose non-fermentable
TMZ: Temozolomide
THCA: Thyroid carcinoma
TCs: Transcription complexes
TFs: Transcription factors
TNBC: Triple-negative breast cancer
TICs: Tumor initiating cells
TME: Tumor microenvironment
UBC: Urothelial bladder cancer
VDR: Vitamin D receptor
